# Coexisting overactive–underactive bladder and detrusor overactivity–underactivity in pelvic organ prolapse

**DOI:** 10.1002/ijgo.14288

**Published:** 2022-06-13

**Authors:** Matteo Frigerio, Marta Barba, Alice Cola, Federico Spelzini, Rodolfo Milani, Stefano Manodoro

**Affiliations:** ^1^ Department of Obstetrics and Gynecology ASST Monza, Ospedale San Gerardo Monza Italy; ^2^ Department of Obstetrics and Gynecology Università degli Studi di Milano‐Bicocca Milan Italy; ^3^ Department of Obstetrics and Gynecology AUSL Romagna, Ospedale Infermi Rimini Italy; ^4^ Department of Obstetrics and Gynecology ASST Santi Paolo e Carlo, Ospedale San Paolo Milan Italy

**Keywords:** coexistent overactive–underactive bladder, coexistent detrusor overactivity–underactivity, pelvic organ prolapse, surgery, underactive bladder, urodynamics

## Abstract

**Objective:**

The coexisting overactive–underactive bladder (COUB) syndrome could be related to the increased urethral resistance caused by severe pelvic organ prolapse (POP). We aimed to evaluate the clinical and urodynamic findings of patients with COUB and/or detrusor overactivity–underactivity (DOU) in a cohort of patients scheduled for POP surgery and the possible risk factors of COUB after surgery.

**Methods:**

This retrospective study analyzed all patients who underwent POP repair between 2008 and 2013, excluding women with a history of pelvic floor surgery. Patients were divided into COUB and non‐COUB according to baseline symptoms and into DOU and non‐DOU based on urodynamic findings. A multivariate model was performed to identify risk factors for COUB symptoms after surgery.

**Results:**

A total of 533 women underwent POP surgery. Preoperatively, patients with COUB had more severe anterior compartment prolapse (Pelvic Organ Prolapse Quantification staging system Aa point, *P* = 0.008) and more frequently had overactive bladder compared with controls (*P* = 0.023). The rate of COUB decreased significantly after surgery. Preoperative opening detrusor pressure resulted as the only independent predictor of postoperative COUB symptoms (*P* = 0.034).

**Conclusion:**

POP is a valid pathogenetic model for COUB development. POP repair induced a significant decrease in COUB symptoms with low opening detrusor pressure resulting as the only independent predictor of postoperative COUB.

## Synopsis

Pelvic organ prolapse is a valid pathogenetic model of coexisting overactive‐underactive bladder syndrome.

## 1 INTRODUCTION

Pelvic organ prolapse (POP) is associated with various lower urinary tract dysfunctions including overactive bladder and voiding symptoms.^1^, ^2^, ^3^ Recently, a new lower urinary tract clinical syndrome has arisen, namely the coexisting overactive–underactive bladder (COUB).^4^ This is defined as a syndrome “characterized by coexisting storage and emptying symptoms in the same patient, without implying any specific urodynamic/functional findings or causative physiology; and that these symptoms are suggestive of urodynamically demonstrable coexistent detrusor overactivity/underactivity, but can be caused by other forms of urethro‐vesical dysfunctions.”^4^

The pathogenesis is believed to be multifactorial, including neurogenic and nonneurogenic factors, with aging playing a major role, inducing progressive decay of neural and/or nonneural functions, including degeneration and biochemical changes caused by cellular damage and apoptosis.^4^ However, the etiopathogenetic process is not well defined. Different mechanisms have been proposed, including: (a) afferent dysfunctions leading to a decrease or a premature start and end of the micturition reflex^5^; (b) the lack of detrusor rest during the filling phase caused by abnormal activation, leading to muscle waste of energy and exhaustion in the next voiding phase^4^; (c) a chronic ischemic‐reperfusion injury caused by blood supply impairment, leading to alteration of myocyte contractile function, patchy denervation, and substitution of the detrusor muscle with fibrous connective^6^; (d) the autonomous contraction of small areas of the detrusor (“micromotions”) during the voiding phase caused by sparse denervation, inducing detrusor overactivity (DO) during storage and inefficient contraction during the voiding phase^7^; and (e) a bladder outlet obstruction–induced remodeling of the detrusor, including initial compensatory hypertrophy and later decompensation with progressive deterioration of bladder function.^8^

The diagnostic criteria of COUB and instrumental findings are not yet established, and definition is the subject of ongoing debates. Specifically, urodynamic correlates of this syndrome are unclear, since urodynamic tests may either show or not show DO and detrusor underactivity (DU). This is, at least in part, caused by the paucity of instrumental studies focusing on the coexistence of DO–underactivity (DOU). Still, urodynamic tests are recommended in the presence of unclear cases or cases not responding to initial treatment.^2^ Interestingly, significant differences in urodynamic findings have been found between patients who have DU without DO and patients who have DU with DO.^9^ This is particularly important, since the characterization of patients with DU and coexisting DO may help to establish reliable diagnostic criteria for COUB. In this context, in 2019 the International Consultation on Incontinence Research Society identified the definition of COUB urodynamic findings as one of their future research priorities. This is pivotal to improve understanding of the population characteristics, the natural evolution, and the proper management of COUB syndrome. This may be even more important in the female population, in whom the link between bladder symptoms and detrusor activity may be less clear.^3^

Since one of the proposed etiopathogenetic mechanisms of COUB and DOU is believed to be the increase of urethral resistance, severe POP may represent a promising model to investigate this new clinical entity in the female population. However, to date, there are no data on the relationship between POP and DOU. As a consequence, we aimed to evaluate the prevalence, clinical features, and urodynamic findings of patients with COUB and/or DOU in a cohort of patients scheduled for POP surgery. Moreover, we investigated the evolution of COUB after POP surgery and the role of possible risk factors.

## 2 MATERIAL AND METHODS

This retrospective study represents a secondary analysis of a previous article focusing on DU in patients with POP.^10^ All consecutive patients who underwent primary POP repair between 2008 and 2013 were analyzed. Patients with a history of previous surgery for pelvic floor disorders were excluded. Preoperative evaluation included a medical interview to investigate the presence of urinary, bowel, bulging, and sexual symptoms. The gynecological examination included POP staging according to the Pelvic Organ Prolapse Quantification (POP‐Q) staging system.^11^ All patients underwent preoperative urodynamic evaluation according to the International Continence Society (ICS) Good Urodynamic Practices and Terms.^12^ Detrusor underactivity was evaluated through the Bladder Contractility Index (BCI) (pDet@Q_max_ + Q_max_ × 5) proposed by Abrams.^13^ A BCI < 100 was considered indicative of DU. Based on baseline symptoms, patients were divided into COUB and non‐COUB groups. According to urodynamic findings, the population was divided into DOU and non‐DOU. All patients underwent native‐tissue repair through vaginal hysterectomy plus high uterosacral ligaments suspension, which represented our standard procedure for POP primary repair in case of menopausal women or fertile‐age women not requiring uterine preservation.^14^, ^15^ Bilateral salpingectomy and/or bilateral oophorectomy were performed—when technically feasible—according to menopausal status, age, adnexal pathology, oncologic risk, and the patient's will. Apex suspension was performed through high uterosacral suspension according to the previously described technique.^16^, ^17^ Additional surgical procedures such as anterior repair and posterior repair were performed when needed. Patients were followed up 1 and 6 months after surgical repair, then annually. Follow‐up visits included a clinical interview to investigate the presence of lower urinary tract symptoms, including storage and voiding symptoms according to ICS terminology.^18^ POP staging according to the POP‐Q was performed at every visit, and a postoperative descent stage II or higher in any compartment or the need for reoperation for POP was considered as objective recurrence. The presence of bulging symptoms was considered a subjective recurrence.

The study was approved by the ethics committee of San Gerardo Hospital in Monza, Italy. Data were entered in the database by one author and double‐checked by another author. Statistical analysis was performed using JMP software version 9.0 (SAS Institute Inc). Continuous data are reported as mean ± standard deviation and noncontinuous variables as absolute (relative) frequency. Differences were tested with Student *t* test for continuous parametric data, Wilcoxon test for continuous nonparametric data, and Pearson chi‐square test for noncontinuous data. A *P* < 0.05 was considered statistically significant. A multivariate model was performed to identify risk factors for COUB symptoms after surgery, using only variables significantly associated with the outcome in the univariate analysis.

## 3 RESULTS

During the study period, a total of 533 women underwent vaginal hysterectomy and high uterosacral ligament vault suspension. Preoperative data, urodynamic evaluation, operative data, and follow‐up were available for 518 patients (dropout rate 2.8%). According to baseline symptoms, 88 (17%) patients were classified as having COUB, while the remaining 430 had non‐COUB. Based on preoperative urodynamic findings, the population was divided into 33 (6.4%) women with DOU and 485 patients without DOU. While the association between the two conditions was statistically significant (*P* = 0.002), the observed proportionate agreement was 81.3%, resulting in only slight agreement according to Cohen κ coefficient (κ = 0.11). No differences between groups were found in terms of population characteristics (Table 1), except for age, which was significantly higher in patients with COUB (65.5 vs. 63.1 years, *P* = 0.049) and DOU (68.2 vs. 63.1 years, *P* = 0.007) compared with controls. Patients with DOU had a higher rate of both overactive bladder syndrome (60.6% vs. 34.4%, *P* = 0.003) and voiding symptoms (63.6% vs. 44.5%, *P* = 0.033) compared with controls (Table 2). Patients with COUB had preoperatively more severe anterior compartment prolapse in terms of point Aa (+1.4 ± 1.4 vs. +0.9 ± 1.5, *P* = 0.008) compared with controls (Table 3). Patients with either COUB or DOU demonstrated substantial similarities in terms of urodynamic findings compared with controls (Table 4). In particular, both groups showed lower BCI (*P* = 0.014 and *P* < 0.001) and maximum flow (*P* = 0.017 and *P* < 0.001), but higher detrusor pressure at maximum cystometric capacity (*P* = 0.025 and *P* < 0.001) and at the beginning of urinary flow (*P* = 0.020 and *P =* 0.016), as well as postvoid residuals both in absolute volumes (*P* = 0.017 and *P* < 0.001) and in the proportion to maximum cystometric capacity (*P* = 0.013 and *P* < 0.001). Moreover, patients with DOU had lower first desire (138.3 vs. 175.3 ml, *P* = 0.024) and maximum cystometric capacity (366.8 vs. 435.5 ml, *P* < 0.001) volumes compared with women without DOU.

**TABLE 1:** Population characteristics

	COUB			DOU		
	Yes	No	*P* value	Yes	No	*P* value
Age, year	65.5 ± 10.4	63.1 ± 10.5	**0.049**	68.2 ± 8.3	63.1 ± 10.5	**0.007**
Menopausal status	84 (95.5%)	390 (90.7%)	0.145	32 (97.0%)	442 (91.1%)	0.245
BMI, kg/m^2^	25.1 ± 3.8	25.0 ± 3.8	0.874	25.8 ± 3.9	25.0 ± 3.8	0.170
Parity	2.2 ± 1.1	2.1 ± 1.0	0.612	2.3 ± 1.4	2.1 ± 1.0	0.723
Operative delivery	18 (20.5%)	62 (14.4%)	0.153	4 (12.1%)	76 (15.7%)	0.585

*Note*: Continuous data are expressed as mean ± standard deviation and noncontinuous data as absolute (relative) frequency. Bold values indicate significance.

Abbreviations: BMI, body mass index; COUB, coexisting overactive–underactive bladder; DOU, detrusor overactivity–underactivity.

**TABLE 2:** Preoperative symptoms

	COUB			DOU		
	Yes, *n* (%)	No, *n* (%)	*P* value	Yes, *n* (%)	No, *n* (%)	*P* value
Stress urinary incontinence	42 (47.7)	180 (37.2)	0.065	10 (30.3)	192 (39.6)	0.290
Overactive bladder syndrome	88 (100)	99 (23.0)	**<0.001**	20 (60.6)	167 (34.4)	**0.003**
Voiding symptoms	88 (100)	149 (34.7)	**<0.001**	21 (63.6)	216 (44.5)	**0.033**
Constipation	33 (37.5)	120 (27.9)	0.072	10 (30.3)	143 (29.5)	0.921
Dyspareunia	11 (12.5)	50 (11.6)	0.817	5 (15.2)	56 (11.6)	0.534

*Note*: Data are expressed as absolute (relative) frequency. Bold values indicate significance.

Abbreviations: COUB, coexisting overactive–underactive bladder; DOU, detrusor overactivity–underactivity.

**TABLE 3:** Preoperative POP‐Q

Points	COUB			DOU		
	Yes	No	*P* value	Yes	No	*P* value
Aa	+1.4 ± 1.4	+0.9 ± 1.5	**0.008**	+1.6 ± 1.4	+0.9 ± 1.5	0.060
Ba	+1.7 ± 1.7	+1.2 ± 1.7	0.152	+1.8 ± 1.4	+1.3 ± 1.7	0.050
C	+0.4 ± 2.7	−0.3 ± 2.9	0.051	−0.4 ± 2.6	−0.2 ± 2.9	0.670
Gh	3.8 ± 0.5	3.7 ± 0.6	**0.039**	3.6 ± 0.6	3.7 ± 0.6	0.634
Pb	2.9 ± 0.4	3.0 ± 0.4	**0.015**	2.9 ± 0.5	3.0 ± 0.4	0.277
Tvl	10.3 ± 1.5	10.5 ± 1.4	0.352	9.9 ± 1.5	10.4 ± 1.4	0.091
Ap	−1.5 ± 1.1	−1.5 ± 1.2	0.859	−1.7 ± 0.9	−1.4 ± 1.2	0.343
Bp	−1.5 ± 1.1	−1.5 ± 1.2	0.987	−1.7 ± 0.9	−1.5 ± 1.2	0.333
D	−4.2 ± 2.8	−4.5 ± 2.8	0.179	−5.2 ± 2.1	−4.4 ± 2.8	0.095

*Note*: Data are expressed as mean ± standard deviation. Bold values indicate significance.

Abbreviations: COUB, coexisting overactive–underactive bladder; DOU, detrusor overactivity–underactivity; POP‐Q, Pelvic Organ Prolapse Quantification staging system.

**TABLE 4:** Preoperative urodynamics

	COUB			DOU		
	Yes	No	*P* value	Yes	No	*P* value
BCI	109.3 ± 48.6	116.8 ± 40.3	**0.014**	79.1 ± 11.8	118.0 ± 42.1	**<0.001**
First desire volume, mL	171.5 ± 82.4	171.6 ± 90.9	0.901	138.3 ± 75.1	175.3 ± 90.0	**0.024**
MCC, mL	422.5 ± 80.4	432.9 ± 83.6	0.313	366.8 ± 93.9	435.5 ± 80.6	**<0.001**
pDet@MCC, cmH_2_O	9.7 ± 7.1	8.4 ± 7.7	**0.025**	14.4 ± 10.1	8.0 ± 7.0	**<0.001**
PDet@Op, cmH_2_O	21.4 ± 11.2	18.4 ± 15.0	**0.020**	24.9 ± 17.1	18.3 ± 13.9	**0.016**
Q_max_, mL/s	16.9 ± 9.9	18.4 ± 8.1	**0.017**	10.5 ± 2.9	18.7 ± 8.4	**<0.001**
pDet@Q_max_, cmH_2_O	24.8 ± 10.2	24.8 ± 12.0	0.873	26.4 ± 10.4	24.7 ± 11.8	0.162
PVR, mL	61.3 ± 98.0	44.2 ± 94.6	**0.017**	80.5 ± 113.1	44.8 ± 93.7	**<0.001**
PVR%	15.6 ± 25.2	10.0 ± 20.7	**0.013**	20.9 ± 27.3	10.3 ± 21.0	**<0.001**
USUI	28 (31.8%)	115 (26.7%)	0.332	10 (30.3%)	133 (27.4%)	0.720

*Note*: Noncontinuous data are expressed as absolute (relative) frequency and continuous data as mean ± standard deviation. Values in bold indicate significance.

Abbreviations: BCI, Bladder Contractility Index; COUB, coexisting overactive–underactive bladder; DOU, detrusor overactivity–underactivity; MCC, maximum cystometric capacity; pDet@MCC, detrusor pressure at maximum cystometric capacity; pDet@Op, opening detrusor pressure; pDet@Q_max_, detrusor pressure at maximum flow; Q_max_, maximum flow; PVR, postvoid residual; PVR%, postvoid residual/maximum cystometric capacity*100; USUI, urodynamic stress urinary incontinence.

Operative data, including operative time and bleeding were similar between groups. Anterior and posterior repair were performed respectively in 87.5% and 72.6% of patients, without differences between groups. No differences were found in terms of transient postoperative urinary retention between groups. At a mean of 33 months of follow‐up, there were no differences in terms of POP‐Q points, objective or subjective recurrence, and reoperation rate. Postoperative symptoms evaluation did not show any difference between groups, except for overactive bladder syndrome, which was higher in preoperative patients with COUB compared with controls (37.5% vs. 25.6%, *P* = 0.023) (Table 5). In particular, postoperative voiding symptoms was similar in both COUB–non‐COUB and DOU–non‐DOU groups. The rate of COUB decreased significantly from 17.0% at baseline to 6.2% postoperatively (*P* < 0.001). Interestingly, neither preoperative COUB nor DOU was a significant risk factor for the presence of COUB symptoms after surgery. Opening detrusor pressure during preoperative workup was the only independent predictor of postoperative COUB symptoms, with an odds ratio of 0.96 for each cmH_2_O‐unit increase (*P* = 0.034).

**TABLE 5:** Postoperative symptoms

	COUB			DOU		
	Yes, *n* (%)	No, *n* (%)	*P* value	Yes, *n* (%)	No, *n* (%)	*P* value
Stress urinary incontinence	17 (19.3)	91 (21.2)	0.698	5 (15.2)	103 (21.2)	0.405
Overactive bladder syndrome	33 (37.5)	110 (25.6)	**0.023**	13 (39.4)	130 (26.8)	0.118
Voiding symptoms	12 (13.6)	70 (16.3)	0.536	3 (9.1)	79 (16.3)	0.273
COUB	4 (4.6)	28 (6.5)	0.485	2 (6.1)	30 (6.2)	0.977
Constipation	26 (29.6)	93 (21.6)	0.108	5 (15.2)	114 (23.5)	0.270
Dyspareunia	6 (6.8)	28 (6.5)	0.916	1 (3.0)	33 (6.8)	0.397
Bulging symptoms	9 (10.2)	21 (4.9)	0.051	1 (3.0)	29 (6.0)	0.483

*Note*: Data are expressed as absolute (relative) frequency. Value in bold indicates signficance.

Abbreviations: COUB, coexisting overactive–underactive bladder; DOU, detrusor overactivity–underactivity.

## 4 DISCUSSION

Up‐to‐date diagnostic criteria of COUB are not yet established, and the urodynamic correlates of this syndrome are poorly understood. Although the definition of COUB itself suggests the correlation with coexistent DOU, it does not rely on urodynamics tests, which are thus not considered mandatory in the diagnostic workup. This uncertainty is exacerbated by the incomplete knowledge about COUB development and the paucity of etiopathogenetic models. One of the proposed models is represented by bladder outlet obstruction. The increase in urethral resistance may involve at the beginning compensatory hypertrophy of detrusor, which may subsequently evolve in decompensation with progressive deterioration of bladder function. While this model is frequently observed with aging in male patients as a result of benign prostatic hyperplasia, this is less frequent in women. However, advanced POP represents a promising model in female patients, and has already been studied for detrusor underactivity.^10^, ^19^, ^20^

With this study we aimed to evaluate the prevalence, clinical features, and urodynamic findings of patients with COUB and/or DOU in a cohort of patients scheduled for POP surgery. Moreover, we investigated the evolution of COUB after POP and the role of possible risk factors. We confirmed the central role of aging in COUB/DOU development since in both conditions the mean age was significantly higher than in controls. This is believed to be related to progressive degeneration and biochemical changes caused by cellular damage and apoptosis, thus impairing both neural and/or nonneural functions.^4^ This was confirmed by previous studies from Gammie^9^ and Stav^21^ and colleagues who reported that both male and female patients with DO and concomitant impaired contractility were significantly older than controls. We also found a 17% rate of COUB in patients with advanced prolapse, which is relatively high. Moreover, patients with COUB had a more severe anterior compartment prolapse in terms of point Aa compared with controls. These findings suggest that POP is a valid pathogenetic model for COUB development in female patients, attributable to its effect on urethral resistance. In particular, an advanced anterior prolapse may cause mechanical bladder outlet obstruction by urethral kinking. In case of long‐lasting obstruction, such as in the case of large and neglected cystocele, detrusor loss of function may be observed.^22^ It has been previously demonstrated that detrusor function impairment is directly related to the severity of anterior compartment prolapse.^10^, ^23^ Evaluation of urodynamic parameters demonstrates in patients with anterior POP stage III or higher, lower maximum flow (Q_max_) and BCI compared with controls.^23^ In our study, urodynamic tests demonstrated a strong relationship between COUB and DOU (*P* = 0.002), and overall substantial similarities in terms of urodynamic findings between the two conditions. In particular, both groups showed high detrusor pressure at maximum cystometric capacity, high opening detrusor pressure with the low Q_max_, and high postvoid residuals. This finding is in agreement with a previous report from Araki, which showed that in patients with POP, RPM is inversely related to detrusor power according to Schafer's nomogram.^24^ Although the COUB definition does not rely on urodynamics, we think––based on these findings––that urodynamic tests can be useful to improve knowledge about this condition, since it presents a well‐defined urodynamic profile. Moreover, while evidence of DOU was not found in all patients with COUB, and the agreement between the two was low, the substantial similarities in urodynamics findings may suggest a continuum between the two, with DOU likely representing the most severe aspect of the detrusor abnormalities in patients with COUB. This hypothesis is consistent with the fact that patients with DOU presented with additional urodynamic alterations, such as low first desire and maximum cystometric capacity volumes. This is consistent with a previous urodynamic study from Gammie,^9^ in which patients with concomitant DU and DO demonstrated lower bladder volumes at first desire to void and lower voided volumes compared with controls.

It has been proposed that treatments which decrease outlet resistance may improve detrusor function unless extensive and irreversible changes in the bladder wall such as loss of muscle tissue and increased collagen deposition have developed.^10^ In our series, prolapse surgical repair resulted in a significant decrease in COUB symptoms from 17.0% to 6.2% (*P* < 0.001), once again confirming that POP‐related bladder outlet obstruction represents an interesting model of COUB pathogenesis. However, not all of the patients recovered from COUB symptoms, with preoperative opening detrusor pressure resulting as the only independent predictor of postoperative COUB symptoms, with an odds ratio of 0.96 for each cmH_2_O‐unit increase (*P* = 0.034). Since opening pressure may be considered as a marker of detrusor function, it may be hypothesized that the more the detrusor is damaged, the less bladder function is likely to recover despite prolapse repair. This suggests the presence of a sort of functional reserve of detrusor contraction. Probably, if the alteration of myocyte contractile function caused by denervation and substitution with fibrous connective is substantial, COUB is not going to recover despite bladder outlet obstruction removal. This hypothesis has been––at least partially––demonstrated in animal models but still lacks confirmation in humans.^25^, ^26^

To the best of our knowledge, this is the first work to evaluate both the prevalence of COAB and DOU in a cohort of patients with POP. Other strengths include the large numerosity of the population and the multimodal evaluation including population characteristics, preoperative symptoms, POP‐Q assessment, and urodynamic evaluation. Limitations include the retrospective study design and the lack of postoperative urodynamic evaluation. Moreover, we were not able to analyze the impact of other risk factors, such as diabetes and neurological disorders.

## 5 CONCLUSIONS

Our study suggests that POP is a valid pathogenetic model for COUB development in female patients, because of its effect on urethral resistance. In particular, we found a prevalence rate of COUB as high as 17.0% in patients with POP, with advanced anterior compartment prolapse representing a significant risk factor. We confirmed the central role of aging in COUB/DOU development. Patients with COUB and DOU demonstrated substantial similarities in terms of urodynamics findings, suggesting a central role of this diagnostic tool in the interpretation and management of COUB. After bladder outlet obstruction removal prompted by POP repair, a significant decrease in COUB symptoms was observed, with low opening detrusor pressure resulting as the only independent predictor of postoperative COUB. This suggests that the detrusor has an important functional reserve, and only in cases of long‐lasting obstruction with extensive fibrous substitution of myocyte, bladder function is not going to recover despite outlet obstruction removal.

## AUTHOR CONTRIBUTIONS

MF: project development, data analysis, data interpretation, article writing, and article revision/approval. MB: project development, data analysis, data interpretation, article writing, and article revision/approval. AC: project development, data analysis, data interpretation, article writing, and article revision/approval. FS: project development, data analysis, data interpretation, article writing, and article revision/approval. RM: project development, data analysis, data interpretation, article writing, and article revision/approval. SM: project development, data analysis, data interpretation, article writing, and article revision/approval.

## CONFLICT OF INTEREST

The authors declare that they have no conflicts of interest.

## Data Availability

Research data are not shared.
